# Very briefly hiding the hand impedes goal-directed arm movements

**DOI:** 10.1177/03010066251389521

**Published:** 2025-10-25

**Authors:** Eli Brenner, Jeroen B.J. Smeets

**Affiliations:** Department of Human Movement Sciences, 1190 Vrije Universiteit Amsterdam, The Netherlands

**Keywords:** pointing/hitting, perception/action, visuomotor delay, stroboscopic illumination, motor control

## Abstract

Seeing the position and motion of one's hand helps guide the hand to objects that one wants to interact with. If the latest available visual information guides the hand at each moment, slightly delaying access to such information should impede performance. We show that increasing the average delay by a few milliseconds, by briefly hiding the hand, does indeed increase the time it takes to reach a target.

One reason why people are so successful at reaching desired movement endpoints is that they continuously reconsider their movements on the basis of the latest information (Brenner & Smeets, 2025). This allows them to take both unexpected deviations in their movements and changes in the environment into account, and to benefit from the judgment of the remaining movement becoming more reliable as the hand approaches the object that they are reaching out for. It is therefore not surprising that movements are less precise when visual information about the moving hand is removed (Carlton, 1981). Doing so forces people to rely on the visual information that was available before the hand was occluded, together with kinaesthetic information about the moving hand, for the duration of the occlusion. Assuming that people always rely on the latest information, periodically occluding vision of the hand for brief periods will increase the effective visuomotor delay, so it might impede goal-directed arm movements.

Periodically occluding vision with shutter glasses impaired ball-catching (Elliott et al., 1994). The impairment was larger when vision was removed for longer periods of time, even when the total time during which vision was absent was constant: the fraction of balls that were caught gradually decreased as the periods during which vision was occluded increased from about 40 to 180 ms. This is in line with the idea that increasing the effective visuomotor delay by periodically occluding vision impedes movements. However, it could also be a consequence of intermittent presentation at low rates disrupting motion perception (which is why stroboscopic illumination is sometimes used for entertainment). Disrupting motion perception forces participants to rely heavily on their experience with gravitational acceleration to anticipate how the ball's motion will proceed. Artificially varying the delay in a cursor-based task also suggested that longer delays impede performance (Brenner et al., 2023), but cursor-based tasks introduce conflicts between the seen and felt motion of the hand: the cursor remains visible for some time at each position, and there is an inevitable delay between the motion of the hand and the cursor. Here, we examine how increasing the effective visuomotor delay by periodically occluding vision of the hand affects performance when the occlusion is so short that it does not disrupt motion perception.

After providing written consent, 20 seated, right-handed participants (17–51years old) tried to tap on targets on a horizontal surface with their right index finger. Their task was to tap on as many targets as possible within 60 s. The targets (blue, 1.8 cm diameter disks) seemed to be on the horizontal surface, below a half-silvered mirror (Figure 1A), but were actually presented on a screen above the mirror. Participants could see their hand and the surface on which they tapped through the half-silvered mirror when the light below the mirror was *on*. This was the case 10% of the time. They could always see the target. We measured their finger movements at 500 Hz (Optotrak 3020) to detect when and where they tapped. Once a tap was detected (based on the vertical deceleration of the finger), a new target appeared, 9 cm from the previous target's position in a random direction (but within a 40.5 cm diameter circular region). Targets were presented on a dark grey background, rather than a black one, to ensure that only the reflected light was visible when the light below the mirror was *off*. The light below the mirror was produced by several strips of light-emitting diodes surrounding the mirror. A light-tight curtain covering the arm prevented participants from looking straight into the light reflected by the (black) surface that they tapped on. The timing of the light *on* and *off* periods was controlled by dedicated hardware manufactured by BitWizard (Delft, The Netherlands). We varied the cycle duration. Although participants noticed the flicker for the longer cycle durations, it was not evident that there were times at which they could not see their hand. Participants also never reported noticing that their finger did not completely occlude the target when they overlapped in space.

**Figure 1.**
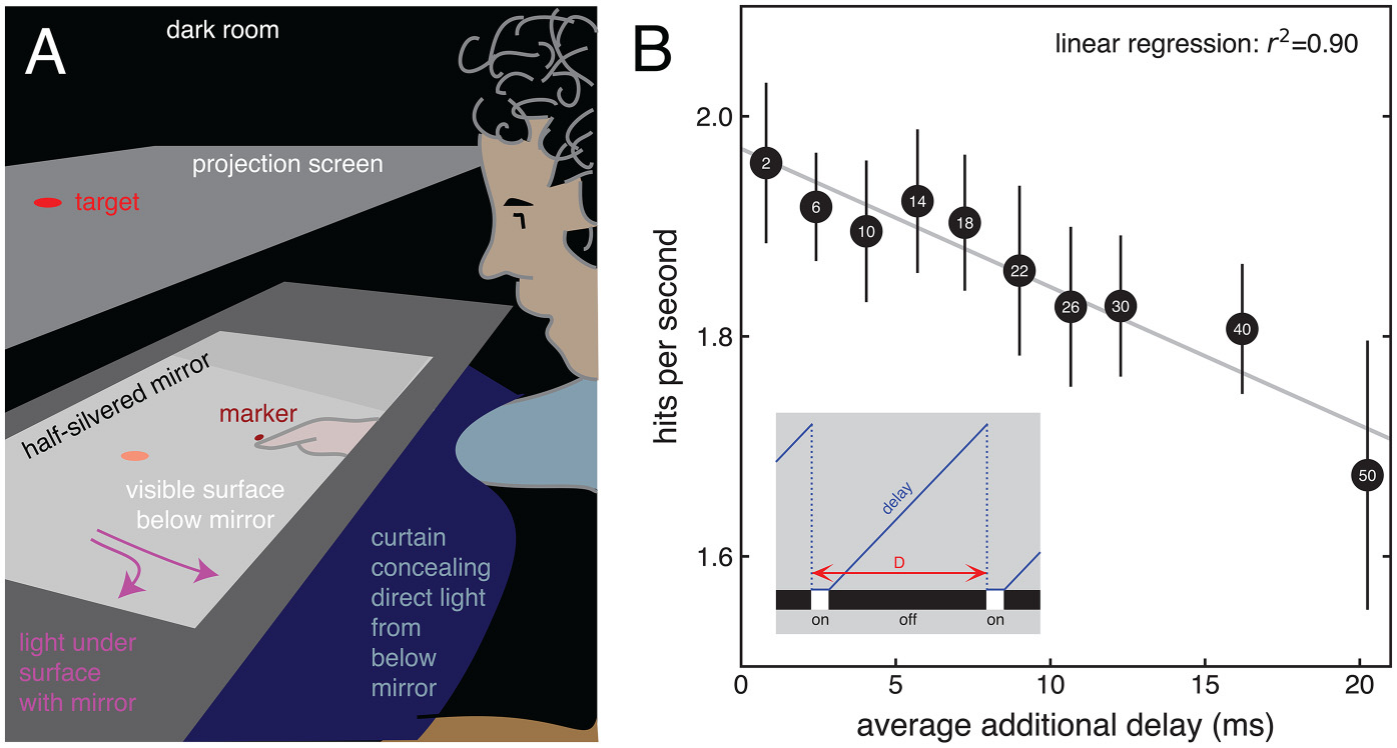
Setup and Results. (A) The target reflected by the half-silvered mirror appeared to lie on a surface that could be seen through the mirror when the light beneath the mirror was *on*. This light was *on* 10% of the time. When it was *on*, participants could see their hand. Their task was to tap the target with their finger as quickly as they could. Once they tapped, the target moved to a new position. (B) The light flickered with cycle durations (*D*) ranging from 2 to 50 ms (values within points; in ms). Whenever the light turns *off*, participants can rely on earlier visual information about the finger's position, but that means using information with an additional delay (see line in inset). The delay gradually increases until the light turns *on* again, so until it reaches 90% of the cycle duration (the time during which the light was *off*). When the hand is visible (light *on*), there is no additional delay. On average, the additional delay when the light is *off* is half the duration of the occlusion (45% of the cycle duration). Thus, overall, the average additional delay is 0.9 × 0.45 × *D*. Fewer targets were hit as the average additional delay increased.

After some practice, each participant performed the task for 10 cycle durations in a semi-random order (each cycle duration was presented first for two participants, second for two participants, and so on). We estimated the time taken to hit the target by dividing the median time between when the target appeared and when a tap was detected (on average 421 ms) by the fraction of targets that were hit (on average 78%). We used the reciprocal of this value as our measure of performance: a number of hits per second. The number of hits decreased as the cycle duration increased. We attribute this to the average additional delay increasing with the cycle duration (Figure 1B). We refer to the delay as being “additional” because it is introduced by our manipulation (in addition to the approximately 100 ms “natural” visuomotor delay). The dependence on the visuomotor delay supports the idea that constantly reconsidering ongoing movements is critical for human proficiency at reaching out for objects.
